# From AI access to perceived learning capability in rural schools: a capital-capability account of pedagogically mediated use

**DOI:** 10.3389/fpsyg.2026.1934154

**Published:** 2026-08-31

**Authors:** Meili Li, Shuai Xu

**Affiliations:** School of Marxism, Sias University, Zhengzhou, China

**Keywords:** artificial intelligence in education, capability approach, educational inequality, pedagogical mediation, perceived AI-supported learning capability, rural education

## Abstract

This explanatory sequential mixed-methods study gave qualitative evidence interpretive priority while using the quantitative strand as supplementary and contextualizing evidence. It examined how family, school, pedagogical, and learner-use conditions were associated with perceived AI-supported learning capability among students in six rural lower-secondary schools in China. The quantitative strand drew on 486 valid student questionnaires, supplemented by contextual questionnaires from 24 teachers, and described sample patterns and selected associations. The qualitative strand comprised 32 semi-structured interviews with students, teachers, parents, and school leaders and provided the primary basis for interpreting how AI-supported learning was experienced and organized. Integrating Bourdieu’s theory of capital with Sen’s capability approach, the study treats mediated conversion as a conceptual account of how AI resources are socially and pedagogically organized, not as a statistically established indirect effect. AI-use frequency and teacher mediation varied across family-resource and home-connectivity conditions. In the supplementary school-fixed-effect regression model, teacher mediation, planned academic AI use, and output-evaluation confidence showed the strongest focal associations with perceived learning capability. Interviews indicated that attempt-feedback-explanation-revision routines supported explanation, verification, revision, and persistence, whereas weakly guided use was often oriented toward rapid completion. The findings distinguish nominal access from perceived capability and identify possible compensatory patterns alongside risks of reproducing existing inequalities. The cross-sectional, self-report design does not establish causal conversion, statistical mediation, school-level inference, or objective achievement gains.

## 1 Introduction

Artificial intelligence (AI) has become a prominent element of educational reform. AI-assisted learning systems are frequently presented as means of personalizing instruction, extending feedback, and compensating for uneven educational provision. Yet access to AI does not automatically generate inclusion. Its educational significance depends on the conditions under which students encounter, interpret, evaluate, and use AI-supported resources. Research has therefore cautioned against treating AI as a neutral or self-sufficient educational good, emphasizing that its educational implications are shaped by pedagogy, institutional capacity, learner judgement, and broader questions concerning which forms of educational thinking should remain human-led (Costa and Murphy, 2025; Holmes and Tuomi, 2022; Varsik and Vosberg, 2024; Yan et al., 2024).

These concerns are especially consequential in rural schooling. Rural students may study in contexts marked by unstable connectivity, constrained school capacity, uneven teacher preparation, fewer specialized learning resources, and differentiated family educational support. Technology may extend the availability of materials, but the ability to incorporate those materials into sustained learning routines is socially distributed. Systematic research on rural technology integration has shown that infrastructure, teacher capacity, organizational support, and local context jointly condition whether digital resources become educationally useful (Mustafa et al., 2024). Evidence from China likewise indicates persistent rural–urban differences in teachers’ digital teaching competence and highlights the importance of ICT skills, data literacy, training, and school-level support (Lin et al., 2023; Wu et al., 2022).

AI intensifies this problem because it places additional interpretive demands on learners. To benefit from adaptive feedback, automated correction, or generative explanations, students must understand the task the system is addressing, evaluate whether an output is credible and curriculum-relevant, identify missing reasoning, and decide when to rely on or challenge machine-generated suggestions. Digital inequality research has long distinguished access from differences in skills, patterns of use, and outcomes (Robinson et al., 2015; van Dijk, 2020). In AI-supported learning, this distinction becomes more consequential because fluent or personalized outputs can create an appearance of usefulness while leaving students uncertain about evidence, reasoning, and disciplinary standards (Lee et al., 2025; Yan et al., 2025).

Existing research has generated valuable evidence concerning AI adoption, digital competence, rural technology integration, and learning outcomes. Less is known, however, about the family, school, pedagogical, and learner-use conditions associated with rural students’ perceived ability to use AI meaningfully for understanding, revision, verification, problem-solving, and independent study. This gap matters because educational equity in the AI era cannot be evaluated through resource provision alone. It also requires attention to whether learners have realistic opportunities to use resources toward educational purposes they value (Sen, 1999; UNESCO, 2025).

This study addresses that gap by developing a capital-capability account of pedagogically mediated AI use in rural schools. Bourdieu’s theory of capital is used to explain unequal entry conditions, including family support, device access, connectivity, and familiarity with school-valued practices. Sen’s capability approach is used to distinguish resource possession from substantive opportunity. The study does not claim to measure capability in its complete normative sense. Instead, it examines perceived AI-supported learning capability: students’ self-reported ability to use AI for valued learning purposes. Pedagogical mediation, planned academic use, evaluative confidence, infrastructure stability, and local norms are conceptualized as conversion conditions that may help explain why similar formal access is associated with different forms of use and perceived benefit.

The term mediated conversion is retained as a conceptual and pedagogical expression rather than a statistical claim. It describes the proposition that resources are interpreted and organized through instructional, social, and learner-level conditions. Because the study is cross-sectional and does not estimate temporally ordered indirect effects, it evaluates associations and participant accounts that are consistent with this proposition; it does not establish statistical mediation, causal transformation, or a definitive mechanism.

The article addresses three research questions:

*RQ1*: How do rural students’ reported access to and use of AI-supported learning vary descriptively across family-resource, home-connectivity, and sampled-school contexts?

*RQ2*: Which pedagogical and learner-use conditions are associated with perceived AI-supported learning capability?

*RQ3*: Under what family and school conditions do quantitative patterns and participant accounts suggest possible compensatory benefits or risks of reproducing existing educational inequalities?

## 2 Literature review and theoretical framework

### 2.1 AI-supported learning and the limits of access-based equity

AI-supported learning includes adaptive learning platforms, automated feedback systems, and generative AI applications that provide explanations, practice recommendations, drafting support, error diagnosis, and problem-solving suggestions. Such tools are commonly associated with personalization, immediate feedback, and expanded access to learning assistance. Major reviews and policy analyses, however, also show that AI can reproduce inequality through uneven connectivity, biased systems, differences in digital competence, and unequal institutional capacity for responsible integration (Holmes and Tuomi, 2022; Varsik and Vosberg, 2024; Vincent-Lancrin and van der Vlies, 2020; Yan et al., 2024).

An access-based account risks assuming that provision creates equivalent benefit. Research on digital inequality instead distinguishes first-level divides in material access from second-level differences in skills and use and third-level differences in outcomes (Robinson et al., 2015; van Dijk, 2020). AI-supported learning adds an epistemic dimension because students must evaluate generated or recommended content rather than merely retrieve information. A chatbot explanation, automated essay comment, or adaptive practice sequence becomes educationally useful only when the learner can connect it to the task, judge its limitations, and incorporate it into a defensible learning process.

Recent research on AI literacy and AI-mediated sense making similarly suggests that benefit depends on evaluative judgement, purposeful interaction, and learning designs that discourage passive dependence. School-focused AI-literacy research distinguishes knowledge, affective orientation, and forms of AI-related thinking, while studies of generative AI highlight the need for verification, metacognition, and epistemic agency (Park, 2025; Silvola et al., 2025; Yan et al., 2025; Zhong and Liu, 2025). The relevant equity issue is therefore not simply whether students use AI, but whether AI use is organized as a learning practice that preserves reasoning and accountability.

For the purposes of this study, AI literacy, planned use, evaluative confidence, perceived learning capability, and learning correlates are treated as conceptually distinct. AI literacy concerns knowledge and critical understanding of AI; planned use concerns how students organize tool use around an academic purpose; output-evaluation confidence concerns perceived ability to assess the relevance or credibility of AI output; perceived AI-supported learning capability concerns perceived ability to employ AI toward valued learning activities; and learning correlates concern reported engagement, confidence, efficiency, and performance. These constructs are expected to be related, but they should not be treated as interchangeable.

### 2.2 Rural schooling and unequal conditions of use

Rural schools are not simply settings with fewer resources. They are educational environments in which material, organizational, and relational conditions shape how available resources are used. Students may encounter intermittent internet access, shared devices, limited technical assistance, less specialized teacher support, and fewer opportunities for informal academic guidance at home. These conditions influence whether technological resources become routine components of learning or remain occasional, unstable, and weakly integrated (Hannan and Eynon, 2025; Mustafa et al., 2024).

Research in China has shown that rural teachers can face constraints in ICT skills, data literacy, digital pedagogical competence, and institutional support (Lin et al., 2023). Teacher- and school-level ICT training are associated with rural teachers’ use of digital educational resources, indicating that professional learning and school organization are consequential for implementation (Wu et al., 2022). Research conducted during large-scale online learning similarly found rural–urban differences in students’ e-learning outcomes and linked those differences to forms of capital such as parental support, teacher support, self-efficacy, and learning motivation (Zhao et al., 2022).

AI-supported learning may consequently contain both compensatory possibilities and reproductive risks. It may provide additional explanations, revision opportunities, and practice feedback where local instructional resources are constrained. Yet AI use also demands judgement, verification, and task alignment. Students with stable connectivity, stronger family support, confident teachers, and established academic routines may therefore be better positioned to benefit. Under rural conditions, inequality may emerge less from the formal presence of tools than from differences in the conditions through which those tools are interpreted, legitimized, and integrated into learning.

### 2.3 Capital, capability, and AI-related educational advantage

Bourdieu’s sociology provides a lens for understanding unequal entry positions in AI-supported learning. Education is a field in which learners bring unequal economic, cultural, and social capital, while institutions recognize some dispositions and practices as more legitimate than others (Bourdieu, 1986, 1990; Bourdieu and Passeron, 1977). Economic capital shapes access to devices and connectivity; cultural capital shapes familiarity with academic language and school-valued strategies; social capital shapes access to guidance; and habitus influences what learners perceive as possible, legitimate, and worth pursuing.

AI does not enter a neutral educational field. Students differ not only in whether they can reach an AI tool but also in whether they can use it in ways that support disciplinary learning. One learner may request a complete answer and copy it, whereas another may use the same system to test an initial interpretation, compare explanations, and revise an argument. These practices are not reducible to individual preference. They are shaped by teacher expectations, family norms, prior experience, assessment pressure, and opportunities to observe academically valued forms of AI use.

Sen’s capability approach complements this account by shifting attention from resources to substantive opportunities. Equivalent resources need not generate equivalent freedoms because individuals face different personal, social, and environmental conversion factors (Sen, 1999). In education, the possession of a device or account does not itself constitute an opportunity to learn if the learner lacks stable access, comprehensible guidance, legitimate time, or the confidence to evaluate outputs.

In Sen’s technical usage, capability refers to a person’s substantive freedom or genuine opportunity to achieve valued functionings, not simply to self-reported competence. The present study therefore distinguishes five related but non-equivalent elements: AI resources, conversion conditions, perceived AI-supported learning capability, enacted learning functionings, and educational outcomes. Resources concern tools, devices, connectivity, and institutional provision. Conversion conditions concern the personal, pedagogical, family, and institutional circumstances through which those resources can be used. Perceived AI-supported learning capability captures students’ reported ability to use AI for understanding, revision, verification, problem-solving, and independent study. Enacted functionings would require evidence of what learners actually do, while educational outcomes would require behavioral, performance, or achievement indicators. The survey measure is consequently treated as a subjective indicator relevant to capability analysis rather than a complete operationalization of capability in Sen’s normative sense.

### 2.4 A capital-capability account of pedagogically mediated AI use

The proposed framework contains four analytically linked layers. Capital and resource conditions include family educational support, home internet stability, device availability, school digital support, and teacher preparedness. Pedagogical and learner-use conditions include teacher mediation, planned academic use, output-evaluation confidence, access to assistance, and the stability of learning routines. The focal empirical construct is perceived AI-supported learning capability. Proximal self-reported learning correlates include academic engagement, learning confidence, task efficiency, and subject performance. These layers are theoretically related, but the present cross-sectional design does not establish a temporally ordered causal sequence.

Pedagogical mediation refers to practices through which teachers frame the purpose of AI use, require students to attempt work before consulting a system, connect feedback to curricular concepts, model verification, and ask learners to justify revisions. Planned academic use concerns whether learners approach AI with a defined learning purpose rather than primarily seeking rapid completion. Output-evaluation confidence concerns perceived ability to judge whether a response is relevant, correct, sufficiently reasoned, and consistent with curricular expectations. Together, these conditions describe how AI use can be made visible, interpretable, and accountable.

**Figure 1:**
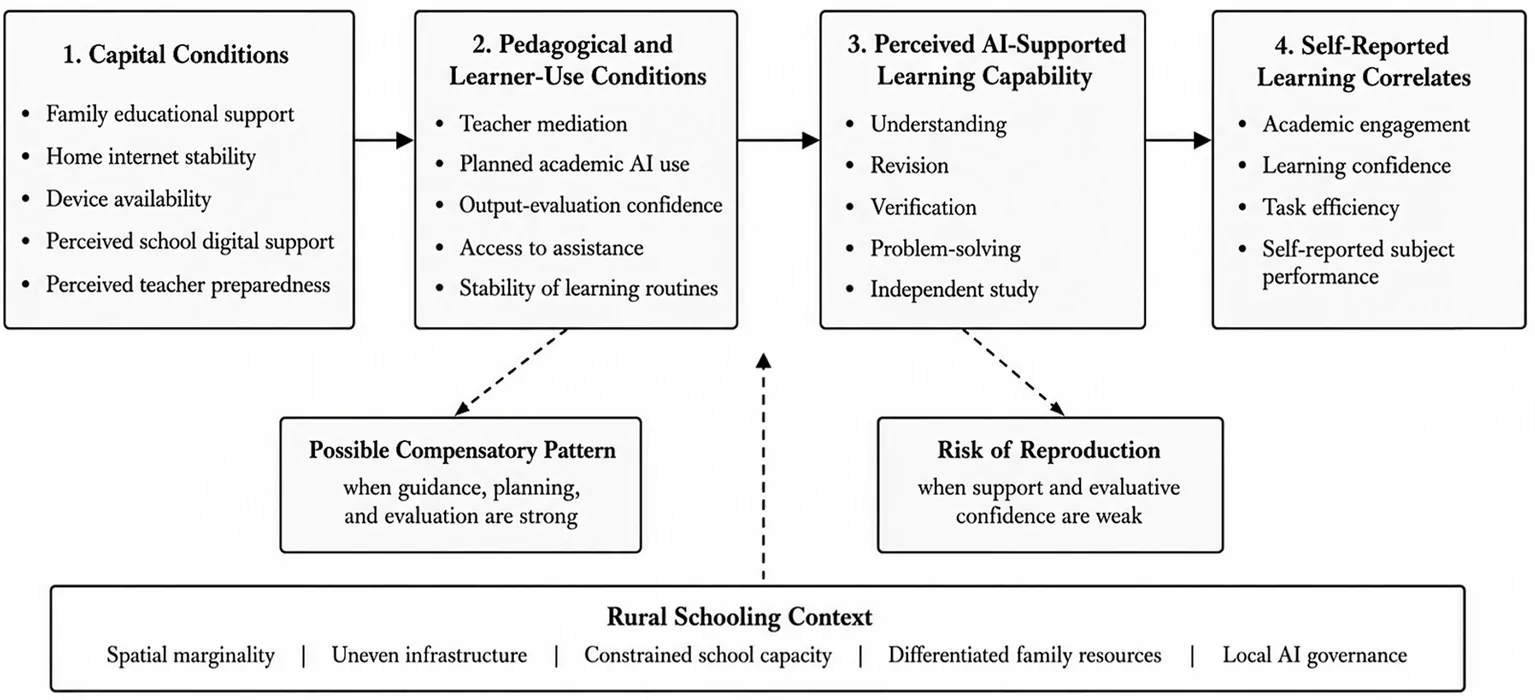
Proposed capital-capability framework for AI-supported learning.

Mediated conversion is used as a conceptual label for this organized relation between resources and use. It is not a claim that the study has identified a statistical mediator. The reduction of a resource coefficient after additional predictors are entered into a regression does not establish an indirect effect, and cross-sectional associations cannot establish temporal sequence. The framework should therefore be understood as a theoretically informed account that can be tested more directly in future longitudinal and intervention research. Figure 1 presents the proposed relationships among resource conditions, pedagogical and learner-use conditions, perceived AI-supported learning capability, and self-reported learning correlates. The framework is conceptual and does not represent statistically established mediation or causal pathways.

## 3 Methodology

### 3.1 Research design

The study used an explanatory sequential mixed-methods design with qualitative priority in interpretation. The survey strand informed subsequent qualitative sampling and provided supplementary descriptive and associational evidence, while the qualitative strand provided the primary basis for interpreting how rural students experienced and made sense of AI-supported learning. The quantitative analyses were therefore not intended to provide population-level causal inference, definitive school-level estimates, or a statistical test of mediation.

This design was appropriate because the research questions concerned both patterned associations and the meanings participants attached to AI use. The study did not seek to estimate treatment effects. It instead aimed to develop a theoretically informed and empirically cautious account of how rural students’ reported AI-related learning opportunities were associated with family, school, pedagogical, and learner-use conditions.

### 3.2 Setting and AI tool contexts

The study was conducted during the 2025 academic year in six rural lower-secondary schools across three county-level regions in China, anonymized as Regions A, B, and C. Two schools were selected from each region to capture variation in local economic conditions, digital infrastructure, and prior participation in digital education initiatives. Eligible schools were officially classified as rural or town-based lower-secondary schools, served surrounding rural communities, and had introduced at least one AI-supported learning resource during the preceding academic year.

The six schools contributed 101, 73, 65, 85, 81, and 81 valid student responses, respectively. The corresponding numbers of contextual teacher questionnaires were 5, 4, 3, 4, 4, and 4. Table 1 reports the distribution of student and teacher respondents across schools. These school summaries document the nested sample structure but are not used to support precise between-school inference because only six schools were included.

**Table 1:** Participant profile.

Panel/group	Value
Panel A. Student survey analytic sample	
Schools	6
Regions	3 county-level regions
Student respondents	486
Grades covered	7–9
Valid response rate	89.7%
Panel B. Respondents by school	
School 1	101 students; 5 teachers
School 2	73 students; 4 teachers
School 3	65 students; 3 teachers
School 4	85 students; 4 teachers
School 5	81 students; 4 teachers
School 6	81 students; 4 teachers
Total	486 students; 24 teachers
Panel C. Qualitative interviews	
Students	16
Teachers	8
Parents	5
School leaders	3
Total interview participants	32

Students and teachers were drawn from survey respondents; parents and school leaders were recruited separately through participating schools.

AI tools fell into three broad categories. Adaptive platforms provided structured practice recommendations, error diagnosis, and stepwise hints. Automated feedback systems supported homework correction, grammar feedback, and writing revision. Generative AI applications provided flexible explanations, vocabulary examples, drafting support, and problem-solving suggestions. The categories were not treated as technically equivalent. They differed in openness of output, curricular structure, and the interpretive demands placed on students. Table 2 summarizes the tool categories, typical functions, use contexts, and reported learning tasks.

**Table 2:** AI tool contexts in the sampled schools.

Tool category	Typical function	Main use context	Learning tasks reported
Adaptive learning platforms	Practice recommendation, error diagnosis, stepwise hints	Mainly school-based; sometimes homework-based	Mathematics practice, English vocabulary, review exercises
Automated feedback systems	Homework correction, grammar feedback, writing suggestions	School- and homework-based	Essay revision, grammar correction, written homework feedback
Generative AI applications	Explanations, drafting support, problem-solving suggestions	Mostly home-based or informal school use	Concept clarification, vocabulary examples, outline generation, checking answers

The study focused on reported educational use rather than brand-specific platform comparison.

### 3.3 Participants and data screening

A total of 542 student questionnaires were returned. Fifty-six questionnaires were excluded before substantive analysis: 28 had more than 20% missing responses on core items, 12 showed invariant or internally inconsistent response patterns, 9 lacked required grade or school information or did not meet the rural lower-secondary sampling criteria, and 7 were removed after parental or student withdrawal. The final student analytic sample comprised 486 respondents, representing an 89.7% valid response rate. Students were enrolled in Grades 7–9. The complete questionnaire items, construct mapping, and scoring procedures are provided in Supplementary Appendix A.

Twenty-four teachers completed contextual questionnaires concerning school infrastructure, AI-related pedagogical practices, professional preparedness, and institutional support. The teacher questionnaires were used for context description and triangulation; they were not entered as 24 additional observations or used as school-level predictors in the student regression model. The small number of teacher respondents within each school precluded precise school-level estimates.

The qualitative sample comprised 32 participants: 16 students, 8 teachers, 5 parents, and 3 school leaders. Student and teacher interviewees were purposively selected from survey respondents using a contrastive sampling strategy. Parents were recruited through participating students and school liaison staff, while leaders were invited because of responsibility for digital-learning implementation. Selection sought variation in access, family support, teacher mediation, school context, tool experience, and perceived educational benefit. Table 1 presents the composition of the quantitative, contextual teacher, and qualitative samples.

### 3.4 Measures and construct boundaries

The student questionnaire covered demographic information, family educational support, home internet stability, device availability, perceived school digital support, perceived teacher preparedness, AI-use frequency, teacher mediation, planned academic AI use, output-evaluation confidence, perceived AI-supported learning capability, and proximal self-reported learning correlates. Items were administered in Chinese. All multi-item constructs used five-point Likert-type response scales.

During the final data audit, the archived binary AI-exposure indicator was found to be inconsistent with the separately recorded AI-use-frequency responses. Because the original routing and coding logic for that binary indicator could not be reconstructed with sufficient certainty from the archived documentation, the indicator was removed from the final analyses rather than retrospectively recoded. AI-use frequency was retained because it represents the directly recorded response categories, ranging from 0 (never) to 3 (daily), with an overall mean of 1.58 (SD = 1.06). AI-related scale responses were recorded for all respondents in the archived analytic dataset, and no skip-coded missing values were present. Because the original routing documentation cannot be reconstructed retrospectively, these scales are interpreted as self-reported perceived capability, confidence, and AI-supported learning orientations in the sampled school context rather than as scores conditional on the removed binary exposure indicator.

Teacher mediation was calculated as the mean of TM1-TM5 and represented the quality and regularity of instructional guidance, including explanation, verification, curriculum connection, and justification of revisions. Its overall mean was 3.31 (SD = 0.88). For the descriptive percentages in Table 3, a teacher-mediation mean of 4.00 or above was classified as high mediation. Perceived school digital support and perceived teacher preparedness were student-reported constructs; the teacher questionnaires remained a separate contextual data source.

**Table 3:** Reported AI use and perceived learning capability by home internet and family support.

Group	*n*	Weekly AI use (%)	High teacher mediation (%)	Perceived capability *M*
Stable home internet	266	62.0	27.1	3.39
Unstable home internet	220	46.4	24.1	2.90
High family support	125	60.8	32.8	3.51
Medium family support	184	59.2	30.4	3.24
Low family support	177	46.3	15.8	2.87

Family-support categories followed the archived grouping variable: low = 1.00–2.50, medium = 2.75–3.50, and high = 3.75–5.00. Because family educational support is the mean of four integer-scored items, possible scale values occur in 0.25 increments. A mean teacher-mediation score of 4.00 or above was classified as high teacher mediation for descriptive purposes. The group comparisons are descriptive and do not establish causal effects.

The focal construct was renamed perceived AI-supported learning capability to align the empirical measure with its self-report character. The six-item index assessed students’ perceived ability to use AI to clarify difficult concepts, revise work, verify responses, solve problems, regulate study, and continue learning independently. It was not treated as a direct measure of students’ complete opportunity sets, observed functionings, or objective achievement.

The instrument was developed in Chinese, reviewed by two bilingual researchers, and pilot-tested with 84 students and 10 teachers in a non-sampled rural school. The pilot informed minor revisions to readability and age appropriateness. The full bilingual item set, item-development basis, response scales, and construct mapping are provided in Supplementary Appendix A. Table 4 summarizes the final measurement properties.

**Table 4:** Measurement reliability and convergent validity.

Construct	Items	Loading range	α	ω	CR	AVE
Family educational support (FS)	4	0.80–0.86	0.89	0.89	0.89	0.67
Home internet stability (HI)	3	0.82–0.84	0.88	0.87	0.87	0.70
Device availability (DA)	3	0.81–0.84	0.87	0.86	0.86	0.68
Perceived school digital support (SDS)	5	0.82–0.85	0.92	0.92	0.92	0.69
Perceived teacher preparedness (TP)	4	0.82–0.85	0.90	0.90	0.90	0.69
Teacher mediation (TM)	5	0.79–0.85	0.92	0.91	0.91	0.67
Planned academic AI use (PAU)	4	0.78–0.84	0.90	0.89	0.89	0.68
Output-evaluation confidence (OEC)	4	0.81–0.84	0.91	0.90	0.90	0.70
Perceived AI-supported learning capability (CF)	6	0.79–0.82	0.93	0.92	0.92	0.64
Academic engagement (ENG)	4	0.80–0.83	0.89	0.88	0.88	0.65
Learning confidence (LC)	4	0.79–0.83	0.90	0.89	0.89	0.66
Task efficiency (TE)	3	0.81–0.82	0.86	0.86	0.86	0.67
Self-reported subject performance (SP)	3	0.79–0.84	0.86	0.86	0.86	0.68

All standardized factor loadings were statistically significant. The focal construct is a perceived-capability indicator and is not presented as a complete measure of capability in Sen’s normative sense. α = Cronbach’s alpha; ω = McDonald’s omega; CR = composite reliability; AVE = average variance extracted.

### 3.5 Measurement validation and common-method considerations

Confirmatory factor analysis was conducted in AMOS 29 using maximum-likelihood estimation (*N* = 486) to evaluate the fit of the hypothesized multifactor measurement structure containing perceived AI-supported learning capability, planned academic use, output-evaluation confidence, engagement, learning confidence, task efficiency, and the other multi-item constructs. The hypothesized multifactor model showed good fit, *χ*^2^(1197) = 1370.03, *p* < 0.001, *χ*^2^/df = 1.15, RMSEA = 0.017, 90% CI [0.012, 0.022], CFI = 0.990, TLI = 0.989, IFI = 0.991, GFI = 0.907, and AGFI = 0.893. The corresponding original AMOS model-fit output is provided in Supplementary Figures S1, S2.

Standardized factor-loading ranges, reliability coefficients, composite reliability values, and average variance extracted values are summarized in Table 4. Supplementary Appendix A provides the final bilingual questionnaire, construct mapping, development basis, and scoring documentation. The verified global CFA output is supplied separately in Supplementary Figures S1, S2.

Most variables were collected from students in the same questionnaire at one time point. Procedural safeguards included anonymous participation, age-appropriate wording, and separation of predictor and correlate sections. Although the multifactor CFA showed good global fit, this does not by itself eliminate concerns about construct overlap, shared-method variance, consistency responding, overlapping wording, or reverse-direction explanations. These limitations are acknowledged explicitly.

### 3.6 Quantitative analytical strategy

The quantitative analysis proceeded in four stages and was used to support, rather than replace, the qualitatively driven interpretation. First, descriptive statistics examined AI-use frequency, teacher mediation, and perceived learning capability across family and school conditions. Second, reliability and measurement-validity analyses evaluated the multi-item indices. Third, an OLS model estimated associations with perceived AI-supported learning capability. Continuous predictors were entered as scale mean scores, while the outcome retained its 1–5 metric; coefficients therefore represent the expected outcome change associated with a one-unit increase in a predictor. Fourth, qualitative and quantitative findings were integrated through contrastive comparison and a joint display.

The regression specification included gender, grade, family educational support, home internet stability, device availability, perceived school digital support, perceived teacher preparedness, teacher mediation, planned academic AI use, output-evaluation confidence, and AI-use frequency. All coefficients were interpreted as concurrent associations rather than evidence of statistical mediation or causal conversion.

The supplementary quantitative model was estimated using ordinary least squares (OLS), with five school indicators included to control for stable mean differences among the six sampled schools. Conventional OLS standard errors, confidence intervals, and *p*-values are reported. Because neither school indicators nor conventional OLS inference corrects for residual within-school dependence, the coefficient-level results are interpreted cautiously as supplementary associational evidence rather than population-level or school-level inference.

A sensitivity model replaced the school indicators with indicators for Regions A, B, and C. The focal estimates remained substantively similar: teacher mediation, *b* = 0.239, SE = 0.046, *p* < 0.001; planned academic AI use, *b* = 0.211, SE = 0.038, *p* < 0.001; and output-evaluation confidence, *b* = 0.182, SE = 0.035, *p* < 0.001. The region-control model had an adjusted *R*^2^ of 0.499 (*N* = 486), compared with 0.509 in the primary school-fixed-effect model. The outcome ICC by school was 0.020. Across the six leave-one-school-out analyses, adjusted *R*^2^ ranged from 0.493 to 0.539; teacher mediation (*b* = 0.192–0.253), planned academic AI use (*b* = 0.210–0.232), and output-evaluation confidence (*b* = 0.169–0.197) remained positive and statistically significant. These checks do not constitute cluster-adjusted coefficient-level inference and are reported only as limited sensitivity analyses.

Categorical access-by-mediation profiles were not retained because categorizing continuous scores required arbitrary thresholds and reduced statistical information. Tool-specific regressions were not estimated because adaptive, automated-feedback, generative-AI, mixed-use, and non-exposure categories did not form sufficiently homogeneous and mutually exclusive groups for a defensible comparison. Compensatory patterns and tool heterogeneity were therefore examined cautiously through descriptive and contrastive qualitative evidence.

Analyses were conducted using IBM SPSS Statistics 29 and AMOS 29. Variance-inflation factors ranged from 1.12 to 2.45, indicating no serious multicollinearity. Standardized residuals ranged from −2.80 to 2.60, and the maximum Cook’s distance was 0.028. Residual and P–P plots did not indicate substantial departures from linearity or normality, and no influential case materially changed the results.

### 3.7 Qualitative data collection and analysis

Interviews were conducted from October to December 2025, approximately 4–6 weeks after completion of the survey. Most interviews were conducted face to face in quiet school meeting rooms or offices, with a small number of online follow-up interviews. Student interviews lasted 20–35 min, and adult interviews lasted 35–55 min. All interviews were conducted in Mandarin Chinese and, with consent, audio-recorded. Separate semi-structured guides were used for students, teachers, parents, and school leaders and addressed everyday AI use, teacher guidance, verification, home-school differences, learning difficulties, implementation, family expectations, policy support, and perceived equity implications.

Research assistants transcribed the recordings verbatim. Analysis was conducted in Chinese using NVivo 14 and proceeded in two cycles. First-cycle open coding identified descriptions of access, guidance, verification, task completion, persistence, family expectations, and practical constraints. Second-cycle theory-informed coding organized these materials around capital and resource conditions, pedagogical and learner-use conditions, perceived learning capability, possible compensatory patterns, and risks of reproduction. Two primary coders with education and sociology backgrounds independently coded approximately 30% of the transcripts. Disagreements were resolved through discussion, with a third researcher reviewing unresolved cases. Contrastive analysis compared students with similar access but different forms of use and students with weaker family support but stronger teacher mediation.

Themes were retained when supported across participants or data sources or when a negative case challenged an emerging interpretation. Individual quotations are used illustratively rather than as evidence of prevalence. Analysis was completed in Chinese; selected quotations were translated by a bilingual researcher and checked through back-translation. The researchers had no formal employment relationship with the participating schools and entered through local education-authority introductions. Reflexive memos were used to consider how prior interest in rural digital inequality could shape interpretation. Sample sufficiency was judged through information power, coverage of contrasting participant roles and schools, and the absence of substantively new themes or codes in the final interviews.

### 3.8 Mixed-methods integration

Integration occurred through contrastive case selection, iterative comparison of survey patterns with interview accounts, and a joint display. Quantitative evidence identified supplementary associations and group patterns; qualitative evidence elaborated how participants understood and enacted AI use under particular conditions. The two strands were not treated as interchangeable. Interview accounts provided plausible contextual explanations but did not convert cross-sectional associations into causal mechanisms.

## 4 Findings

### 4.1 Routine use and unequal conditions

RQ1 was addressed descriptively through comparisons of AI-use frequency, weekly use, teacher mediation, and perceived learning capability across household-resource, connectivity, and sampled-school contexts. These comparisons were not treated as inferential tests of population differences. Across the 486 valid student respondents, the mean AI-use frequency was 1.58 (SD = 1.06) on the 0–3 scale. Approximately 54.9% of the sample reported weekly or daily use. Routine use and high teacher mediation varied across household conditions; no binary AI-exposure rate is interpreted in the final analysis.

Stable home internet was associated descriptively with higher weekly use, high teacher mediation, and perceived learning capability. Students with high family support showed a similar pattern. These contrasts are descriptive because the number of schools was small and the quantitative strand served a supplementary contextualizing function. Table 3 presents the family- and connectivity-related comparisons.

The interview accounts illustrate how formal provision could remain educationally fragile. A Grade 8 student with shared-device access explained, “Sometimes the system works, but sometimes the signal is too slow. When it stops, I just go back to the workbook” (Student 06). A teacher in a lower-support school similarly noted, “We have the platform, but teachers use it differently. Some use it only before exams, and some do not know how to connect it with the lesson” (Teacher 03). These accounts illustrate why tool availability can overstate routine educational access.

### 4.2 Resource conditions, pedagogical mediation, and perceived capability

Table 5 reports the complete coefficients from the supplementary primary school-fixed-effect model and the region-control sensitivity model. In the primary model, teacher mediation (*b* = 0.228, SE = 0.046, *p* < 0.001), planned academic AI use (*b* = 0.222, SE = 0.038, *p* < 0.001), and output-evaluation confidence (*b* = 0.185, SE = 0.035, *p* < 0.001) were positively associated with perceived AI-supported learning capability. The primary model had an adjusted *R*^2^ of 0.509 (*N* = 486).

**Table 5:** Complete associations with perceived AI-supported learning capability.

Predictor	Primary school-FE model b (SE)	95% CI	Region-control sensitivity b (SE)	95% CI
Female	−0.078 (0.056)	[−0.188, 0.032]	−0.086 (0.056)	[−0.196, 0.024]
Grade 8	−0.043 (0.069)	[−0.179, 0.093]	−0.042 (0.070)	[−0.179, 0.094]
Grade 9	0.014 (0.067)	[−0.118, 0.145]	0.025 (0.067)	[−0.107, 0.157]
Family educational support	0.004 (0.037)	[−0.068, 0.076]	0.000 (0.037)	[−0.073, 0.072]
Home internet stability	0.122*** (0.036)	[0.051, 0.193]	0.124*** (0.037)	[0.052, 0.196]
Device availability	0.103* (0.040)	[0.024, 0.182]	0.100* (0.040)	[0.021, 0.180]
Perceived school digital support	0.053 (0.042)	[−0.031, 0.136]	0.068 (0.043)	[−0.015, 0.152]
Perceived teacher preparedness	0.069 (0.048)	[−0.026, 0.164]	0.055 (0.049)	[−0.041, 0.150]
Teacher mediation	0.228*** (0.046)	[0.139, 0.318]	0.239*** (0.046)	[0.148, 0.329]
Planned academic AI use	0.222*** (0.038)	[0.148, 0.296]	0.211*** (0.038)	[0.137, 0.285]
Output-evaluation confidence	0.185*** (0.035)	[0.117, 0.253]	0.182*** (0.035)	[0.114, 0.250]
AI-use frequency	0.020 (0.029)	[−0.037, 0.078]	0.027 (0.029)	[−0.030, 0.085]
School 2	−0.179 (0.094)	[−0.364, 0.005]	—	—
School 3	−0.363*** (0.097)	[−0.554, −0.172]	—	—
School 4	−0.168 (0.090)	[−0.345, 0.008]	—	—
School 5	−0.040 (0.091)	[−0.219, 0.139]	—	—
School 6	−0.269** (0.092)	[−0.449, −0.089]	—	—
Region B	—	—	−0.178** (0.069)	[−0.313, −0.043]
Region C	—	—	−0.078 (0.067)	[−0.210, 0.054]
Adjusted R^2^	0.509	—	0.499	—
OLS F	30.567***	—	35.458***	—
df	17, 468	—	14, 471	—
N	486	—	486	—

Continuous predictors were entered as scale mean scores, while the outcome retained its 1–5 metric. Conventional OLS standard errors are reported in parentheses. School 1 was the reference category in the primary model, and Region A was the reference category in the sensitivity model. The primary model included five school fixed-effect indicators, whereas the sensitivity model replaced school indicators with region indicators. The outcome ICC and leave-one-school-out analyses are supplementary sensitivity checks and should not be interpreted as correcting for residual dependence within schools. *p < 0.05, **p < 0.01, ***p < 0.001.

The focal coefficients changed only slightly when region indicators replaced school fixed effects. This stability supports the descriptive robustness of the association pattern within the sampled schools, but it does not demonstrate statistical mediation, temporal conversion, cluster-adjusted inference, or causal displacement of resource effects.

The qualitative evidence helps interpret these associations. In a mathematics class characterized by strong mediation, students attempted a problem before consulting platform feedback and then wrote a sentence identifying the source of error. A student from a lower-support household explained, “First we try it ourselves. Then the platform shows the wrong step. The teacher asks us to say why it is wrong before we do the next one” (Student 11). The educational sequence was therefore attempt, feedback, explanation, and revision rather than immediate answer retrieval.

A contrasting student had stable internet and a personal laptop but described frequent completion-oriented generative-AI use: “I paste my draft and ask it to make it better. I usually use the answer because it is faster” (Student 04). The contrast does not prove that teacher mediation caused a different outcome, but it illustrates why frequency alone can provide an incomplete account of perceived learning capability.

Complete coefficient-level *p*-values, omnibus model statistics, the outcome ICC, and leave-one-school-out results are reported in Supplementary Appendix B.

### 4.3 Possible compensatory patterns and risks of reproduction

The mixed-methods evidence suggested possible compensatory patterns when school mediation supported students whose home educational resources were weaker. Several students described using teacher-structured AI feedback to revisit explanations, identify types of error, and continue working without immediate adult help. One teacher required students to classify feedback as identifying a calculation error, concept error, or careless error before revising a solution (Teacher 05). A student in the same school said that the routine helped her “know what kind of mistake it is, not only the answer” (Student 13).

These cases are consistent with a compensatory interpretation, but the study does not establish that AI reduced inequality. Such a claim would require longitudinal comparison, a valid access-by-mediation interaction, or an intervention design. The present evidence supports the narrower conclusion that structured school mediation can create meaningful reported learning opportunities for some students with weaker home support.

A contrasting pattern involved access without evaluative support. Some students described using AI to complete tasks quickly without reconstructing the reasoning behind an answer. Others abandoned tools when interfaces were confusing or connectivity unstable. A Grade 9 student described an adaptive platform as “too many buttons and reports” and said he “spent more time clicking than learning” (Student 09). A second student said that a generative response often “sounds correct” while leaving her unable to explain why it worked (Student 02). These accounts suggest a risk of reproducing differences in learning practice when access is not accompanied by interpretive support.

Norms of legitimate use also formed part of the conversion environment. Teachers worried that assistance could become substitution; parents sometimes associated typing or automated correction with weakened discipline; and students alternately positioned AI as a shortcut, a tutor, or a source to be checked. Such expectations influenced whether AI use was encouraged, hidden, questioned, or sustained.

### 4.4 Tool heterogeneity and interpretive burden

Tool-specific regressions were not estimated because the available tool-use categories did not form sufficiently well-defined, mutually exclusive groups for a defensible quantitative comparison. Tool heterogeneity was therefore examined through contextual and qualitative evidence rather than through an under-specified two-group model.

Qualitative and contextual evidence nevertheless indicated meaningful differences in interpretive burden. Generative AI produced fluent, flexible responses that students could find difficult to verify. A student using generative AI for physics homework stated, “It gives a final answer, but if I do not know the formula, I cannot tell whether it is right” (Student 07). Adaptive platforms provided more structured sequences but could still become confusing when dashboards were complex or disconnected from classroom reasoning.

These accounts support a differentiated rather than unitary understanding of AI. Generative systems may place greater demands on verification and source evaluation, while adaptive and automated-feedback systems require careful curriculum alignment and instructional orchestration. The evidence is exploratory and does not establish that one tool category is more equitable or educationally effective than another.

### 4.5 Mixed-methods integration

Table 6 integrates the principal quantitative patterns with corresponding participant accounts and cautious mixed-methods interpretations.

**Table 6:** Mixed-methods joint display.

Quantitative pattern	Qualitative elaboration	Cautious interpretation
Teacher mediation, planned use, and evaluation confidence had the largest focal coefficients in the school-fixed-effect model.	Teachers described attempt-feedback-explanation-revision routines and explicit output checking.	Classroom routines were consistent with a pedagogical role in supporting perceived learning capability.
AI-use frequency was not statistically significant in the full model.	Students reporting similar frequency described different completion- or verification-oriented practices.	Frequency alone provided an incomplete account of perceived learning capability.
Students with stable internet and stronger support reported higher weekly use and perceived capability.	Connectivity interruptions, shared devices, and uneven teacher integration limited routine use.	Nominal provision did not ensure stable or educationally organized access.

The joint display integrates associations with participant accounts. Qualitative evidence offers plausible contextual explanations but does not verify causal mechanisms.

## 5 Discussion

### 5.1 AI equity as a problem of access and pedagogically organized use

The findings suggest that AI equity in rural schooling cannot be evaluated through provision alone. Routine use and high teacher mediation were unevenly distributed across resource conditions. In the supplementary school-fixed-effect model, teacher mediation, planned academic use, and output-evaluation confidence had the largest associations with perceived learning capability, whereas use frequency alone provided a weaker account of meaningful use. These quantitative associations are interpreted as supplementary to the qualitative evidence rather than as population-level or school-level inference.

This pattern extends digital inequality research by drawing attention to a possible conversion divide: differences between learners who can incorporate AI resources into purposeful, evaluative practices and those for whom access remains unstable, completion-oriented, or difficult to interpret. The concept remains provisional because the study measures perceived capability rather than complete opportunity sets or long-term functionings. It nevertheless adds specificity to the distinction among material access, practices of use, and educationally consequential benefit (Robinson et al., 2015; van Dijk, 2020).

The results complicate both optimistic and pessimistic accounts. AI can extend opportunities to revisit explanations and obtain feedback, particularly where adult assistance is limited (Holmes and Tuomi, 2022; Varsik and Vosberg, 2024). Yet its value depends on whether learners can connect outputs to curriculum, reconstruct reasoning, and recognize when human guidance is required.

Some students from lower-support households described meaningful benefit under structured guidance, suggesting that schools may create compensatory opportunities by making AI use sequenced and accountable. Such accounts should not be equated with demonstrated reductions in inequality; the more defensible conclusion is that pedagogical organization may shape how resource differences are experienced in learning.

### 5.2 Mediated conversion as a conceptual account

The theoretical contribution is a conceptual account linking unequal capital and resource conditions with perceived AI-supported learning capability. Bourdieu explains unequal entry positions in resources, educational familiarity, recognized dispositions, and access to guidance. Sen clarifies why possession of a resource does not itself constitute a substantive educational opportunity. The analytical space between possession and opportunity is therefore where pedagogical and social organization matters.

Mediated conversion names that analytical space without claiming that the present models estimate an indirect effect. Teacher mediation, planned use, evaluation confidence, and infrastructure were associated with perceived capability, while participant accounts illustrated how routines and norms organized tool use. Longitudinal and intervention studies are needed to test temporal and causal versions of the framework.

The findings also refine the relationship between capability and AI literacy. AI literacy is commonly framed as technical understanding, critical evaluation, and ethical awareness (Ng et al., 2023; Park, 2025; Prilop et al., 2025; Zhong and Liu, 2025). The interviews indicate that evaluative practices are also pedagogically produced: students learned to question outputs when tasks required comparison, explanation, and justification.

This point connects the framework to epistemic agency. Efficiency does not necessarily produce understanding. Students described greater benefit when AI was used to test explanations, locate errors, and revise thinking rather than retrieve completed answers, consistent with scholarship prioritizing verification, metacognition, and epistemic responsibility (Silvola et al., 2025; Yan et al., 2025).

### 5.3 Compensatory possibilities and risks of reproduction

AI-supported learning offered possible compensatory opportunities when teacher mediation supported students with weaker home resources. Students described revisiting explanations, correcting errors, and persisting when adult assistance was unavailable. The interpretation remains provisional until research demonstrates changes in observed opportunities or outcomes relative to appropriate comparison groups.

Reproductive risks were also visible. Students with strong material access but weak guidance sometimes described rapid completion without reconstructing reasoning. Generative AI was especially challenging when fluent language concealed uncertainty or omitted steps. Related research has raised concerns about cognitive offloading and uncritical dependence, although the present study does not demonstrate cognitive decline (Lee et al., 2025; Tian and Zhang, 2025).

These risks were not technologically predetermined. Students reporting stronger mediation more often described checking, comparing, and revising outputs. Inequality may therefore be reorganized around access to evaluative routines and legitimate guidance, rather than around devices alone.

Tool heterogeneity further complicates equity judgments. Adaptive systems can provide structured sequences but become burdensome when interfaces are complex or poorly aligned with lessons. Generative systems offer flexible explanations but demand stronger evaluation and reconstruction of reasoning. The relevant question is which tools work for which learners under which pedagogical conditions.

### 5.4 Implications for rural AI policy and school practice

First, evaluation of rural AI initiatives should move beyond counts of devices, platforms, accounts, or logins. Monitoring should also consider connectivity stability, teacher mediation, verification opportunities, curriculum alignment, and students’ understanding of legitimate use.

Second, professional development should focus on pedagogical design rather than tool demonstration. Teachers need practical routines that require students to attempt tasks, consult AI, verify the response, and justify revision.

Third, rural initiatives should prioritize low-bandwidth, low-burden, and curriculum-aligned systems. Technical sophistication is not equivalent to educational suitability when unstable connections or complex interfaces consume attention without increasing opportunity.

Fourth, students need explicit, age-appropriate instruction in verification, including comparison with textbooks, identification of unsupported claims, reconstruction of omitted steps, and recognition of when teacher or peer confirmation is necessary.

Finally, schools and families need shared expectations that distinguish assistance, revision, verification, and substitution. Locally negotiated guidance should address acceptable purposes, disclosure, privacy, and the reasoning for which students remain responsible.

## 6 Conclusion

This study examined associations among AI access, family and school conditions, pedagogical guidance, learner-use practices, and perceived AI-supported learning capability in six rural lower-secondary schools in China. Access alone provided an incomplete account of reported learning opportunity. Teacher mediation, planned academic use, and output-evaluation confidence showed the largest associations with perceived capability, while interviews distinguished verification-oriented from shortcut-oriented use.

The study contributes a capital-capability account of pedagogically mediated AI use. Bourdieu explains unequal entry positions, while Sen clarifies why equivalent resources need not provide equivalent opportunities. Mediated conversion refers here to the social and pedagogical organization of resource use, not to a statistically established indirect effect, and the focal construct is defined as perceived learning capability rather than capability in its complete normative sense.

Rural AI equity should therefore be evaluated through more than tool availability. Teacher preparation, verification-oriented routines, stable infrastructure, and locally appropriate governance may all shape meaningful use. Longitudinal research using observations, student work, platform records, objective achievement measures, and larger school samples is required to determine whether the reported associations correspond to sustained changes in capability and functioning.

## 7 Limitations and future research

Several limitations should be noted. First, the cross-sectional design does not establish temporal order or causal effects. Resource conditions, mediation, perceived capability, and learning correlates were measured concurrently; therefore, reverse-direction and omitted-variable explanations remain possible.

Second, the study examines perceived AI-supported learning capability rather than capability in the full Senian sense. The measure captures students’ perceived ability to use AI for valued learning activities but does not directly assess opportunity sets, observed functionings, reasoning quality, or objective achievement.

Third, most quantitative variables were collected through the same student questionnaire. Although the multifactor CFA showed good global fit, shared-method variance, construct overlap, and self-report bias may have influenced the observed associations. The archived binary AI-exposure indicator was removed from final analysis because its coding could not be reconciled with the recorded AI-use-frequency responses without imposing a retrospective correction rule.

Fourth, students were nested within only six schools. School indicators, ICC assessment, and leave-one-school-out analyses improved transparency, but the limited number of schools prevents stable school-level inference, conventional multilevel modeling, or cluster-adjusted population inference.

Finally, the teacher questionnaires were used for contextual triangulation rather than school-level estimation. Future research should combine larger teacher samples, classroom observations, infrastructure audits, longitudinal designs, and pre-specified statistical sensitivity procedures to examine how AI-related opportunities develop over time.

## Data Availability

The original contributions presented in the study are included in the article/Supplementary material, further inquiries can be directed to the corresponding author.
