# Supplementary material for: From AI access to perceived learning capability in rural schools: a capital-capability account of pedagogically mediated use
Source: Front Psychol. 2026 Aug 31;17:1934154. doi: 10.3389/fpsyg.2026.1934154 (PMC13572710; doi:10.3389/fpsyg.2026.1934154)
Supplement: Supplementary file 1 [file Supplementary_file_3.docx]

CFA.amw
- Analysis Summary
- Notes for Group
- Variable Summary
- Parameter Summary
- Notes for Model
- Estimates
- Modification Indices
- Minimization History
- Model Fit
- Execution Time

Group number 1

Default model

Model Fit Summary

CMIN

Model	NPAR	CMIN	DF	P	CMIN/DF
Default model	181	1370.031	1197	.000	1.145
Saturated model	1378	.000	0		
Independence model	52	19531.042	1326	.000	14.729

RMR, GFI

Model	RMR	GFI	AGFI	PGFI
Default model	.086	.907	.893	.788
Saturated model	.000	1.000		
Independence model	.374	.133	.099	.128

Baseline Comparisons

Model	NFI Delta1	RFI rho1	IFI Delta2	TLI rho2	CFI
Default model	.930	.922	.991	.989	.990
Saturated model	1.000		1.000		1.000
Independence model	.000	.000	.000	.000	.000

Parsimony-Adjusted Measures

Model	PRATIO	PNFI	PCFI
Default model	.903	.839	.894
Saturated model	.000	.000	.000
Independence model	1.000	.000	.000

NCP

Model	NCP	LO 90	HI 90
Default model	173.031	85.778	268.569
Saturated model	.000	.000	.000
Independence model	18205.042	17756.386	18660.128

FMIN

Model	FMIN	F0	LO 90	HI 90
Default model	2.825	.357	.177	.554
Saturated model	.000	.000	.000	.000
Independence model	40.270	37.536	36.611	38.474

CFA.amw
- Analysis Summary
- Notes for Group
- Variable Summary
- Parameter Summary
- Notes for Model
- Estimates
- Modification Indices
- Minimization History
- Model Fit
- Execution Time

Group number 1

Default model

Parsimony-Adjusted Measures

Model	PRATIO	PNFI	PCFI
Default model	.903	.839	.894
Saturated model	.000	.000	.000
Independence model	1.000	.000	.000

NCP

Model	NCP	LO 90	HI 90
Default model	173.031	85.778	268.569
Saturated model	.000	.000	.000
Independence model	18205.042	17756.386	18660.128

FMIN

Model	FMIN	F0	LO 90	HI 90
Default model	2.825	.357	.177	.554
Saturated model	.000	.000	.000	.000
Independence model	40.270	37.536	36.611	38.474

RMSEA

Model	RMSEA	LO 90	HI 90	PCLOSE
Default model	.017	.012	.022	1.000
Independence model	.168	.166	.170	.000

AIC

Model	AIC	BCC	BIC	CAIC
Default model	1732.031	1776.443	2489.735	2670.735
Saturated model	2756.000	3094.120	8524.595	9902.595
Independence model	19635.042	19647.802	19852.725	19904.725

ECVI

Model	ECVI	LO 90	HI 90	MECVI
Default model	3.571	3.391	3.768	3.663
Saturated model	5.682	5.682	5.682	6.380
Independence model	40.485	39.560	41.423	40.511

HOELTER

Model	HOELTER .05	HOELTER .01
Default model	453	466
Independence model	36	36
