# Supplementary material for: From AI access to perceived learning capability in rural schools: a capital-capability account of pedagogically mediated use
Source: Front Psychol. 2026 Aug 31;17:1934154. doi: 10.3389/fpsyg.2026.1934154 (PMC13572710; doi:10.3389/fpsyg.2026.1934154)
Supplement: Supplementary file 1 [file Supplementary_file_1.docx]

**Supplementary Appendix A**

**Questionnaire, Construct Mapping, Scoring, and Archived Measurement Documentation**

# A1. Administration, scoring, and analytic scope

The questionnaire was administered in Chinese. All multi-item constructs used a five-point Likert-type response scale (1 = does not describe me at all; 5 = describes me completely). Construct scores were calculated as the mean of the retained items. No items were reverse scored. Items removed during pilot testing are not included in the final instrument. The English wording below is a reporting translation checked by bilingual researchers; the Chinese wording is the administered version.

AI-use frequency was retained in the final analysis as a single observed variable using the archived response coding 0 = never, 1 = less than weekly, 2 = weekly, and 3 = daily. The archived binary AI-exposure indicator was removed from the final manuscript after the data audit because its coding could not be reconciled with the recorded frequency responses without imposing a retrospective correction rule. All retained multi-item scale responses included in the final analysis were available for the 486 valid cases.

Across the 52 indicators used in the CFA, the final analytic dataset contained no missing item responses; therefore, the CFA used the complete N = 486 analytic sample and no item-level imputation was required.

# A2. Archived CFA specification and verified global fit

Confirmatory factor analysis was conducted in AMOS 29 using maximum-likelihood estimation. The archived model contained 52 observed indicators loading on 13 latent factors. Each indicator loaded on its intended factor only; no cross-loadings and no correlated residuals were estimated in the archived model.

| Indicator | Verified value |
| --- | --- |
| Analytic sample | N = 486 |
| Estimation method | Maximum likelihood (ML) |
| Observed indicators | 52 |
| Latent factors | 13 |
| Free parameters (NPAR) | 181 |
| Degrees of freedom | 1197 |
| χ² | 1370.031 |
| p | < .001 |
| χ²/df | 1.145 |
| RMSEA | .017 |
| RMSEA 90% CI | [.012, .022] |
| PCLOSE | 1.000 |
| CFI | .990 |
| TLI | .989 |
| IFI | .991 |
| GFI | .907 |
| AGFI | .893 |

Parameter-count note. A fully correlated set of 13 latent factors contains 78 unique factor covariances. The archived AMOS specification freely estimated 77 factor covariances; the covariance between task efficiency (TE) and self-reported subject performance (SP) was not freely estimated and therefore remained fixed at zero in the archived model. This accounts for the reported NPAR = 181 and df = 1197. The final manuscript does not assign substantive meaning to this restriction. No post-hoc correlated residuals or cross-loadings were introduced. The original AMOS model-fit output is supplied separately in Supplementary Figures S1 and S2.

# A3. Construct mapping and development basis

| Construct | Code | Items | Development basis |
| --- | --- | --- | --- |
| Family educational support | FS | 4 | Adapted from research on family educational capital and parental involvement in rural China (e.g., Zhao et al., 2022). |
| Home internet stability | HI | 3 | Adapted from digital-divide and rural ICT-access research and contextualised for home learning. |
| Device availability | DA | 3 | Adapted from digital-divide and rural ICT-access research and contextualised for home learning. |
| Perceived school digital support | SDS | 5 | Adapted from research on school digital support and rural technology integration (e.g., Wu et al., 2022; Lin et al., 2023). |
| Perceived teacher preparedness | TP | 4 | Adapted from teacher digital-competence and school-support research (e.g., Wu et al., 2022; Lin et al., 2023). |
| Teacher mediation | TM | 5 | Developed for this study from the pedagogical-mediation framework and research on teacher-guided technology use. |
| Planned academic AI use | PAU | 4 | Developed for this study to capture goal-directed and planned academic use. |
| Output-evaluation confidence | OEC | 4 | Developed from AI-literacy and critical-evaluation research (e.g., Park, 2025; Yan et al., 2025). |
| Perceived AI-supported learning capability | CF | 6 | Developed from the capability approach and the study definition of valued AI-supported learning activities (Sen, 1999). |
| Academic engagement | ENG | 4 | Adapted from student-engagement measures and contextualised for AI-supported learning. |
| Learning confidence | LC | 4 | Adapted from learning-confidence measures and contextualised for AI-supported learning. |
| Task efficiency | TE | 3 | Adapted from self-reported learning-efficiency measures and contextualised for AI-supported learning. |
| Self-reported subject performance | SP | 3 | Adapted from self-reported learning-outcome measures and contextualised for AI-supported learning. |

# A4. Measurement evidence summary

Standardised loading ranges below are taken directly from the archived AMOS output and are shown to three decimals. The main manuscript reports the same ranges rounded to two decimals. Reliability and convergent-validity coefficients match Table 3 of the final manuscript.

| Construct | Items | Loading range | α | ω | CR | AVE |
| --- | --- | --- | --- | --- | --- | --- |
| Family educational support (FS) | 4 | 0.799-0.858 | .89 | .89 | .89 | .67 |
| Home internet stability (HI) | 3 | 0.819-0.844 | .88 | .87 | .87 | .70 |
| Device availability (DA) | 3 | 0.806-0.844 | .87 | .86 | .86 | .68 |
| Perceived school digital support (SDS) | 5 | 0.821-0.845 | .92 | .92 | .92 | .69 |
| Perceived teacher preparedness (TP) | 4 | 0.818-0.847 | .90 | .90 | .90 | .69 |
| Teacher mediation (TM) | 5 | 0.788-0.849 | .92 | .91 | .91 | .67 |
| Planned academic AI use (PAU) | 4 | 0.782-0.837 | .90 | .89 | .89 | .68 |
| Output-evaluation confidence (OEC) | 4 | 0.811-0.844 | .91 | .90 | .90 | .70 |
| Perceived AI-supported learning capability (CF) | 6 | 0.786-0.822 | .93 | .92 | .92 | .64 |
| Academic engagement (ENG) | 4 | 0.797-0.826 | .89 | .88 | .88 | .65 |
| Learning confidence (LC) | 4 | 0.786-0.833 | .90 | .89 | .89 | .66 |
| Task efficiency (TE) | 3 | 0.810-0.823 | .86 | .86 | .86 | .67 |
| Self-reported subject performance (SP) | 3 | 0.788-0.839 | .86 | .86 | .86 | .68 |

| Factor | Item | Standardised loading |
| --- | --- | --- |
| FS | FS1 | 0.814 |
| FS | FS2 | 0.858 |
| FS | FS3 | 0.808 |
| FS | FS4 | 0.799 |
| HI | HI1 | 0.843 |
| HI | HI2 | 0.844 |
| HI | HI3 | 0.819 |
| DA | DA1 | 0.844 |
| DA | DA2 | 0.818 |
| DA | DA3 | 0.806 |
| SDS | SDS1 | 0.845 |
| SDS | SDS2 | 0.838 |
| SDS | SDS3 | 0.824 |
| SDS | SDS4 | 0.821 |
| SDS | SDS5 | 0.828 |
| TP | TP1 | 0.823 |
| TP | TP2 | 0.836 |
| TP | TP3 | 0.847 |
| TP | TP4 | 0.818 |
| TM | TM1 | 0.788 |
| TM | TM2 | 0.838 |
| TM | TM3 | 0.827 |
| TM | TM4 | 0.790 |
| TM | TM5 | 0.849 |
| PAU | PAU1 | 0.782 |
| PAU | PAU2 | 0.831 |
| PAU | PAU3 | 0.837 |
| PAU | PAU4 | 0.835 |
| OEC | OEC1 | 0.844 |
| OEC | OEC2 | 0.811 |
| OEC | OEC3 | 0.842 |
| OEC | OEC4 | 0.837 |
| CF | CF1 | 0.786 |
| CF | CF2 | 0.806 |
| CF | CF3 | 0.797 |
| CF | CF4 | 0.822 |
| CF | CF5 | 0.804 |
| CF | CF6 | 0.792 |
| ENG | ENG1 | 0.826 |
| ENG | ENG2 | 0.799 |
| ENG | ENG3 | 0.798 |
| ENG | ENG4 | 0.797 |
| LC | LC1 | 0.816 |
| LC | LC2 | 0.822 |
| LC | LC3 | 0.833 |
| LC | LC4 | 0.786 |
| TE | TE1 | 0.817 |
| TE | TE2 | 0.823 |
| TE | TE3 | 0.810 |
| SP | SP1 | 0.836 |
| SP | SP2 | 0.839 |
| SP | SP3 | 0.788 |

# A5. Item-level standardised factor loadings

# A6. Latent-factor correlation matrix

Values are standardised latent-factor correlations estimated in the final CFA model using maximum-likelihood estimation. The TE-SP covariance was not freely estimated in the archived model and is shown as 0* to document the fixed-zero specification.

| Factor | FS | HI | DA | SDS | TP | TM | PAU | OEC | CF | ENG | LC | TE | SP |
| --- | --- | --- | --- | --- | --- | --- | --- | --- | --- | --- | --- | --- | --- |
| FS | 1.000 |  |  |  |  |  |  |  |  |  |  |  |  |
| HI | 0.282 | 1.000 |  |  |  |  |  |  |  |  |  |  |  |
| DA | 0.536 | 0.610 | 1.000 |  |  |  |  |  |  |  |  |  |  |
| SDS | 0.213 | 0.064 | 0.152 | 1.000 |  |  |  |  |  |  |  |  |  |
| TP | 0.241 | 0.037 | 0.160 | 0.750 | 1.000 |  |  |  |  |  |  |  |  |
| TM | 0.245 | 0.214 | 0.221 | 0.591 | 0.732 | 1.000 |  |  |  |  |  |  |  |
| PAU | 0.407 | 0.383 | 0.459 | 0.120 | 0.155 | 0.215 | 1.000 |  |  |  |  |  |  |
| OEC | 0.309 | 0.149 | 0.216 | 0.342 | 0.436 | 0.397 | 0.295 | 1.000 |  |  |  |  |  |
| CF | 0.326 | 0.387 | 0.424 | 0.383 | 0.438 | 0.531 | 0.468 | 0.471 | 1.000 |  |  |  |  |
| ENG | 0.318 | 0.293 | 0.288 | 0.354 | 0.423 | 0.593 | 0.326 | 0.367 | 0.700 | 1.000 |  |  |  |
| LC | 0.239 | 0.261 | 0.295 | 0.354 | 0.380 | 0.467 | 0.352 | 0.572 | 0.732 | 0.504 | 1.000 |  |  |
| TE | 0.277 | 0.338 | 0.348 | 0.157 | 0.183 | 0.284 | 0.537 | 0.324 | 0.615 | 0.389 | 0.453 | 1.000 |  |
| SP | 0.164 | 0.115 | 0.165 | 0.309 | 0.359 | 0.391 | 0.175 | 0.287 | 0.465 | 0.544 | 0.394 | 0* | 1.000 |

* Fixed at zero by the archived AMOS specification; not freely estimated. The largest freely estimated latent correlation was .750 (SDS-TP). Additional discriminant-validity analyses beyond the CFA model were not included because the final manuscript limits measurement claims to the evidence directly supported by the final measurement model.

# A7. Variance documentation from archived AMOS output

The following tables report the unstandardised latent-factor variances and indicator residual variances from the archived AMOS output.

| Factor | Variance | Factor | Variance |
| --- | --- | --- | --- |
| FS | 0.634 | HI | 0.749 |
| DA | 0.675 | SDS | 0.789 |
| TP | 0.711 | TM | 0.733 |
| PAU | 0.745 | OEC | 0.726 |
| CF | 0.591 | ENG | 0.594 |
| LC | 0.604 | TE | 0.703 |
| SP | 0.690 |  |  |

**Indicator residual variances**

| Item | Residual term | Variance | Item | Residual term | Variance |
| --- | --- | --- | --- | --- | --- |
| FS4 | e1 | 0.359 | FS3 | e2 | 0.367 |
| FS2 | e3 | 0.288 | FS1 | e4 | 0.380 |
| HI3 | e5 | 0.367 | HI2 | e6 | 0.295 |
| HI1 | e7 | 0.317 | DA3 | e8 | 0.364 |
| DA2 | e9 | 0.345 | DA1 | e10 | 0.315 |
| SDS5 | e11 | 0.363 | SDS4 | e12 | 0.349 |
| SDS3 | e13 | 0.344 | SDS2 | e14 | 0.326 |
| SDS1 | e15 | 0.308 | TP4 | e16 | 0.352 |
| TP3 | e17 | 0.318 | TP2 | e18 | 0.311 |
| TP1 | e19 | 0.357 | TM5 | e20 | 0.283 |
| TM4 | e21 | 0.373 | TM3 | e22 | 0.304 |
| TM2 | e23 | 0.293 | TM1 | e24 | 0.371 |
| PAU4 | e25 | 0.323 | PAU3 | e26 | 0.302 |
| PAU2 | e27 | 0.310 | PAU1 | e28 | 0.404 |
| OEC4 | e29 | 0.310 | OEC3 | e30 | 0.310 |
| OEC2 | e31 | 0.390 | OEC1 | e32 | 0.299 |
| CF6 | e33 | 0.352 | CF5 | e34 | 0.306 |
| CF4 | e35 | 0.290 | CF3 | e36 | 0.307 |
| CF2 | e37 | 0.304 | CF1 | e38 | 0.339 |
| ENG4 | e39 | 0.340 | ENG3 | e40 | 0.342 |
| ENG2 | e41 | 0.371 | ENG1 | e42 | 0.299 |
| LC4 | e43 | 0.373 | LC3 | e44 | 0.300 |
| LC2 | e45 | 0.312 | LC1 | e46 | 0.289 |
| TE3 | e47 | 0.368 | TE2 | e48 | 0.352 |
| TE1 | e49 | 0.344 | SP3 | e50 | 0.422 |
| SP2 | e51 | 0.314 | SP1 | e52 | 0.338 |

# A8. Final bilingual questionnaire items

The item wording below reproduces the final retained multi-item questionnaire content. The Chinese wording is the administered version; the English wording is the reporting translation.

**Family educational support (FS)**

Development basis: Adapted from research on family educational capital and parental involvement in rural China (e.g., Zhao et al., 2022).

| Item | Chinese item (administered) | English reporting translation |
| --- | --- | --- |
| FS1 | 我的父母/家人经常关心我的学习情况。 | My parents or family members regularly take an interest in my learning. |
| FS2 | 父母/家人会主动帮助我解决学习中遇到的困难。 | My parents or family members actively help me address difficulties in my learning. |
| FS3 | 家里有适合我学习的安静环境。 | My home provides a quiet environment suitable for study. |
| FS4 | 父母/家人对我的学业有较高的期望。 | My parents or family members have high expectations for my academic work. |

**Home internet stability (HI)**

Development basis: Adapted from digital-divide and rural ICT-access research and contextualised for home learning.

| Item | Chinese item (administered) | English reporting translation |
| --- | --- | --- |
| HI1 | 我家里的网络连接稳定，很少断网。 | My home internet connection is stable and is rarely interrupted. |
| HI2 | 在家学习时，我能顺畅地使用需要网络的在线资源。 | When studying at home, I can use online resources smoothly. |
| HI3 | 网络速度足够支持我正常使用学习平台。 | The internet speed is sufficient for me to use learning platforms normally. |

**Device availability (DA)**

Development basis: Adapted from digital-divide and rural ICT-access research and contextualised for home learning.

| Item | Chinese item (administered) | English reporting translation |
| --- | --- | --- |
| DA1 | 我有自己专用的学习设备（如平板或电脑）。 | I have a device dedicated to my learning, such as a tablet or computer. |
| DA2 | 我可以方便地使用手机或电脑进行学习。 | I can conveniently use a mobile phone or computer for learning. |
| DA3 | 家里有足够的设备供我学习使用（无共享冲突）。 | My household has enough devices for my learning without conflicts over sharing. |

**Perceived school digital support (SDS)**

Development basis: Adapted from research on school digital support and rural technology integration (e.g., Wu et al., 2022; Lin et al., 2023).

| Item | Chinese item (administered) | English reporting translation |
| --- | --- | --- |
| SDS1 | 学校提供了良好的数字学习设备和网络。 | My school provides good digital-learning equipment and internet access. |
| SDS2 | 学校有专门的技术支持人员帮助解决问题。 | My school has dedicated technical-support staff who help solve problems. |
| SDS3 | 学校经常组织我们使用数字学习工具。 | My school regularly organises opportunities for us to use digital-learning tools. |
| SDS4 | 学校有足够的AI或数字学习资源。 | My school has sufficient AI or digital-learning resources. |
| SDS5 | 学校领导重视数字教学设施建设。 | School leaders attach importance to developing digital-teaching facilities. |

**Perceived teacher preparedness (TP)**

Development basis: Adapted from teacher digital-competence and school-support research (e.g., Wu et al., 2022; Lin et al., 2023).

| Item | Chinese item (administered) | English reporting translation |
| --- | --- | --- |
| TP1 | 我的老师熟练掌握数字教学工具的使用。 | My teachers are proficient in using digital-teaching tools. |
| TP2 | 老师能够有效地指导我们使用AI学习工具。 | My teachers can guide us effectively in using AI learning tools. |
| TP3 | 老师对AI在教学中的应用有充分准备。 | My teachers are well prepared to apply AI in teaching. |
| TP4 | 老师能解答我们在使用AI工具时遇到的问题。 | My teachers can answer questions that arise when we use AI tools. |

**Teacher mediation (TM)**

Development basis: Developed for this study from the pedagogical-mediation framework and research on teacher-guided technology use.

| Item | Chinese item (administered) | English reporting translation |
| --- | --- | --- |
| TM1 | 老师要求我们在使用AI前先自己尝试解答。 | My teacher requires us to attempt the task ourselves before using AI. |
| TM2 | 老师会指导我们如何验证AI给出的答案。 | My teacher guides us in how to verify answers provided by AI. |
| TM3 | 老师帮助我们将AI反馈与课本知识联系起来。 | My teacher helps us connect AI feedback with knowledge from the textbook. |
| TM4 | 老师要求我们解释修改后的答案为什么更好。 | My teacher asks us to explain why a revised answer is better. |
| TM5 | 老师会示范如何正确使用AI辅助学习。 | My teacher demonstrates how to use AI appropriately to support learning. |

**Planned academic AI use (PAU)**

| Item | Chinese item (administered) | English reporting translation |
| --- | --- | --- |
| PAU1 | 我使用AI主要是为了更好地理解学习内容。 | I use AI mainly to understand learning content better. |
| PAU2 | 我有明确的学习目标时才会使用AI工具。 | I use AI tools when I have a clear learning goal. |
| PAU3 | 我使用AI是为了完成具体的学习任务，而不是随意聊天。 | I use AI to complete specific learning tasks rather than for casual conversation. |
| PAU4 | 我会提前计划好使用AI来辅助哪部分学习。 | I plan in advance which part of my learning AI will support. |

Development basis: Developed for this study to capture goal-directed and planned academic use.

**Output-evaluation confidence (OEC)**

Development basis: Developed from AI-literacy and critical-evaluation research (e.g., Park, 2025; Yan et al., 2025).

| Item | Chinese item (administered) | English reporting translation |
| --- | --- | --- |
| OEC1 | 我能判断AI给出的答案是否与课本知识一致。 | I can judge whether an answer provided by AI is consistent with textbook knowledge. |
| OEC2 | 我有信心评估AI回答的正确性和完整性。 | I am confident in evaluating the accuracy and completeness of an AI response. |
| OEC3 | 我知道什么时候应该相信AI的输出，什么时候需要核对。 | I know when an AI output can be relied on and when it needs to be checked. |
| OEC4 | 我能发现AI回答中可能存在的错误或遗漏。 | I can identify possible errors or omissions in AI responses. |

**Perceived AI-supported learning capability (CF)**

Development basis: Developed from the capability approach and the study definition of valued AI-supported learning activities (Sen, 1999).

| Item | Chinese item (administered) | English reporting translation |
| --- | --- | --- |
| CF1 | 我能使用AI帮助我理解困难的概念。 | I can use AI to help me understand difficult concepts. |
| CF2 | 我能使用AI有效修改和完善我的作业。 | I can use AI to revise and improve my schoolwork effectively. |
| CF3 | 我能使用AI验证自己的答案是否正确。 | I can use AI to verify whether my own answers are correct. |
| CF4 | 我能使用AI辅助解决学习中的问题。 | I can use AI to support problem solving in my learning. |
| CF5 | 我能使用AI规划和调节自己的学习。 | I can use AI to plan and regulate my learning. |
| CF6 | 使用AI后，我更有能力独立继续学习。 | With AI support, I am better able to continue learning independently. |

**Academic engagement (ENG)**

Development basis: Adapted from student-engagement measures and contextualised for AI-supported learning.

| Item | Chinese item (administered) | English reporting translation |
| --- | --- | --- |
| ENG1 | 我在学习时能保持较高的注意力。 | I can maintain a high level of attention while learning. |
| ENG2 | 我积极参与课堂和AI辅助的学习活动。 | I participate actively in classroom and AI-supported learning activities. |
| ENG3 | 我对当前的学习内容感兴趣。 | I am interested in the learning content I am currently studying. |
| ENG4 | 我愿意投入额外时间完成学习任务。 | I am willing to spend additional time completing learning tasks. |

**Learning confidence (LC)**

Development basis: Adapted from learning-confidence measures and contextualised for AI-supported learning.

| Item | Chinese item (administered) | English reporting translation |
| --- | --- | --- |
| LC1 | 我相信自己有能力学好当前科目。 | I believe I am capable of learning my current subjects well. |
| LC2 | 我对自己的学习进步感到满意。 | I am satisfied with my progress in learning. |
| LC3 | 我有信心应对学习中的挑战。 | I am confident that I can deal with challenges in learning. |
| LC4 | 使用AI后，我的学习自信心有所提升。 | Using AI has increased my confidence in learning. |

**Task efficiency (TE)**

| Item | Chinese item (administered) | English reporting translation |
| --- | --- | --- |
| TE1 | 使用AI后，我完成作业的速度更快。 | With AI support, I complete schoolwork more quickly. |
| TE2 | AI帮助我更高效地复习知识点。 | AI helps me review learning content more efficiently. |
| TE3 | 我能用更少的时间掌握相同内容。 | I can master the same content in less time. |

Development basis: Adapted from self-reported learning-efficiency measures and contextualised for AI-supported learning.

**Self-reported subject performance (SP)**

Development basis: Adapted from self-reported learning-outcome measures and contextualised for AI-supported learning.

| Item | Chinese item (administered) | English reporting translation |
| --- | --- | --- |
| SP1 | 我本学期成绩比以前有所进步。 | My academic performance this term has improved compared with before. |
| SP2 | 我在主要科目上的表现良好。 | I perform well in my core subjects. |
| SP3 | 我对自己的考试成绩感到满意。 | I am satisfied with my examination results. |

# References cited in this appendix

Lin, R., Chu, J., Yang, L., Lou, L., Yu, H., & Yang, J. (2023). What are the determinants of rural-urban divide in teachers' digital teaching competence? Empirical evidence from a large sample. Humanities and Social Sciences Communications, 10, 422. https://doi.org/10.1057/s41599-023-01933-2

Park, J. (2025). A systematic literature review of generative artificial intelligence (GenAI) literacy in schools. Computers and Education: Artificial Intelligence, 9, 100487. https://doi.org/10.1016/j.caeai.2025.100487

Sen, A. (1999). Development as freedom. Alfred A. Knopf.

Wu, D., Yang, X., Yang, W., Lu, C., & Li, M. (2022). Effects of teacher- and school-level ICT training on teachers' use of digital educational resources in rural schools in China: A multilevel moderation model. International Journal of Educational Research, 111, 101910. https://doi.org/10.1016/j.ijer.2021.101910

Yan, L., Pammer-Schindler, V., Mills, C., Nguyen, A., & Gašević, D. (2025). Beyond efficiency: Empirical insights on generative AI's impact on cognition, metacognition and epistemic agency in learning. British Journal of Educational Technology, 56(5), 1675-1685. https://doi.org/10.1111/bjet.70000

Zhao, L., Cao, C., Li, Y., & Li, Y. (2022). Determinants of the digital outcome divide in e-learning between rural and urban students: Empirical evidence from the COVID-19 pandemic based on capital theory. Computers in Human Behavior, 130, 107177. https://doi.org/10.1016/j.chb.2021.107177
