# Supplementary material for: From AI access to perceived learning capability in rural schools: a capital-capability account of pedagogically mediated use
Source: Front Psychol. 2026 Aug 31;17:1934154. doi: 10.3389/fpsyg.2026.1934154 (PMC13572710; doi:10.3389/fpsyg.2026.1934154)
Supplement: Supplementary file 2 [file Supplementary_file_2.docx]

**Supplementary Appendix B**

**Data Audit, Complete Regression Results, and Sensitivity Checks**

# B1. Data-audit note on the archived binary AI-exposure indicator

The archived binary AI-exposure indicator was not retained in the final manuscript because it was not fully consistent with the separately recorded AI-use-frequency responses. Rather than imposing a retrospective correction rule, the final analysis removed the binary indicator and retained AI-use frequency as the directly recorded observed variable. The cross-tabulation below documents the archived inconsistency for transparency.

| Archived binary AI-exposure indicator | Never | Less than weekly | Weekly | Daily | Total |
| --- | --- | --- | --- | --- | --- |
| 0 = no exposure | 55 | 54 | 54 | 25 | 188 |
| 1 = exposure | 44 | 66 | 97 | 91 | 298 |
| Total | 99 | 120 | 151 | 116 | 486 |

Note. AI-use frequency was coded 0 = never, 1 = less than weekly, 2 = weekly, and 3 = daily. The binary exposure variable was removed from final analyses and descriptive interpretations.

# B2. Complete primary school-fixed-effect regression results

The outcome was perceived AI-supported learning capability. Conventional OLS standard errors, confidence intervals, and p-values are reported. School 1 was the reference category. Continuous predictors were entered as scale mean scores.

| Predictor | b | SE | 95% CI | p |
| --- | --- | --- | --- | --- |
| Female | -.078 | .056 | [-.188, .032] | .164 |
| Grade 8 | -.043 | .069 | [-.179, .093] | .539 |
| Grade 9 | .014 | .067 | [-.118, .145] | .839 |
| Family educational support | .004 | .037 | [-.068, .076] | .904 |
| Home internet stability | .122 | .036 | [.051, .193] | < .001 |
| Device availability | .103 | .040 | [.024, .182] | .011 |
| Perceived school digital support | .053 | .042 | [-.031, .136] | .215 |
| Perceived teacher preparedness | .069 | .048 | [-.026, .164] | .156 |
| Teacher mediation | .228 | .046 | [.139, .318] | < .001 |
| Planned academic AI use | .222 | .038 | [.148, .296] | < .001 |
| Output-evaluation confidence | .185 | .035 | [.117, .253] | < .001 |
| AI-use frequency | .020 | .029 | [-.037, .078] | .486 |
| School 2 | -.179 | .094 | [-.364, .005] | .057 |
| School 3 | -.363 | .097 | [-.554, -.172] | < .001 |
| School 4 | -.168 | .090 | [-.345, .008] | .061 |
| School 5 | -.040 | .091 | [-.219, .139] | .660 |
| School 6 | -.269 | .092 | [-.449, -.089] | .003 |

# B3. Region-control sensitivity regression results

This sensitivity model replaced the five school indicators with two region indicators. Region A was the reference category.

| Predictor | b | SE | 95% CI | p |
| --- | --- | --- | --- | --- |
| Female | -.086 | .056 | [-.196, .024] | .124 |
| Grade 8 | -.042 | .070 | [-.179, .094] | .543 |
| Grade 9 | .025 | .067 | [-.107, .157] | .708 |
| Family educational support | .000 | .037 | [-.073, .072] | .990 |
| Home internet stability | .124 | .037 | [.052, .196] | < .001 |
| Device availability | .100 | .040 | [.021, .180] | .013 |
| Perceived school digital support | .068 | .043 | [-.015, .152] | .107 |
| Perceived teacher preparedness | .055 | .049 | [-.041, .150] | .262 |
| Teacher mediation | .239 | .046 | [.148, .329] | < .001 |
| Planned academic AI use | .211 | .038 | [.137, .285] | < .001 |
| Output-evaluation confidence | .182 | .035 | [.114, .250] | < .001 |
| AI-use frequency | .027 | .029 | [-.030, .085] | .354 |
| Region B | -.178 | .069 | [-.313, -.043] | .010 |
| Region C | -.078 | .067 | [-.210, .054] | .245 |

# B4. Conventional OLS omnibus model statistics

The omnibus statistics below correspond to the conventional OLS models reported in Tables B2 and B3. They are not cluster-adjusted tests.

| Model | R² | Adjusted R² | OLS F | df1 | df2 | p | N |
| --- | --- | --- | --- | --- | --- | --- | --- |
| Primary school-FE model | .526 | .509 | 30.567 | 17 | 468 | < .001 | 486 |
| Region-control sensitivity model | .513 | .499 | 35.458 | 14 | 471 | < .001 | 486 |

# B5. Leave-one-school-out sensitivity checks

Each row reports the primary model after omitting one school. The outcome ICC by school was .020. Because only six schools were sampled, these analyses are limited sensitivity checks and do not represent cluster-adjusted coefficient-level inference.

| Omitted school | Adjusted R² | Teacher mediation b | p |
| --- | --- | --- | --- |
| S1 | .506 | .234 | < .001 |
| S2 | .501 | .241 | < .001 |
| S3 | .504 | .209 | < .001 |
| S4 | .504 | .243 | < .001 |
| S5 | .493 | .192 | < .001 |
| S6 | .539 | .253 | < .001 |
| Omitted school | Adjusted R² | Planned academic AI use b | p |
| S1 | .506 | .223 | < .001 |
| S2 | .501 | .210 | < .001 |
| S3 | .504 | .232 | < .001 |
| S4 | .504 | .230 | < .001 |
| S5 | .493 | .215 | < .001 |
| S6 | .539 | .225 | < .001 |
| Omitted school | Adjusted R² | Output-evaluation confidence b | p |
| S1 | .506 | .191 | < .001 |
| S2 | .501 | .186 | < .001 |
| S3 | .504 | .169 | < .001 |
| S4 | .504 | .187 | < .001 |
| S5 | .493 | .197 | < .001 |
| S6 | .539 | .179 | < .001 |

# B6. Final model specification

Outcome: perceived AI-supported learning capability, retained on its 1-5 scale. Primary predictors and controls: gender, grade, family educational support, home internet stability, device availability, perceived school digital support, perceived teacher preparedness, teacher mediation, planned academic AI use, output-evaluation confidence, and AI-use frequency. Reference categories: male, Grade 7, School 1, and Region A. Primary estimator: OLS with conventional standard errors. Sensitivity checks: region-control model, outcome ICC, and leave-one-school-out analysis. Software: IBM SPSS Statistics 29.
